# Prognosis of hospital‐acquired pneumonia/ventilator‐associated pneumonia with *Stenotrophomonas maltophilia* versus *Klebsiella pneumoniae* in intensive care unit: A retrospective cohort study

**DOI:** 10.1111/crj.13537

**Published:** 2022-08-31

**Authors:** Shuping Chen, Dongdong Zou

**Affiliations:** ^1^ Intensive Care Unit Shanghai Jiao Tong University Affiliated Sixth People's Hospital Shanghai China; ^2^ Neurosurgery Department Shanghai Jiao Tong University Affiliated Sixth People's Hospital Shanghai China

**Keywords:** hospital‐acquired pneumonia, *Klebsiella*, prognosis, *S. maltophilia*, ventilator‐associated pneumonia

## Abstract

**Introduction:**

We collected data on ventilator‐associated pneumonia (VAP) and hospital‐acquired pneumonia (HAP) induced by *Stenotrophomonas maltophilia* (SM) and *Klebsiella pneumoniae* (KP) and compared differences between two bacteria in mortality, duration of ventilator use, length of hospital stay, and risk factors for infection.

**Objectives:**

This study aimed to evaluate the prognosis and to find risk factors of SM‐HAP/VAP versus KP‐HAP/VAP in the intensive care unit (ICU).

**Methods:**

This retrospective cohort study included patients admitted to the ICU between June 2019 and June 2021 and diagnosed with SM‐HAP/VAP or KP‐HAP/VAP. The primary outcome was 28‐day mortality.

**Results:**

Ninety‐two HAP/VAP patients (48 with SM‐HAP/VAP and 44 with KP‐HAP/VAP) were included. The 28‐day mortality was 16.7% (8/48 patients) in SM‐HAP/VAP and 15.9% (7/44 patients) in KP‐HAP/VAP (*P* = 0.922). After adjustment for potential confounders, the hazard ratios for 28‐day mortality in SM‐HAP/VAP were 1.3 (95% CI:0.5–3.7), 1.0 (95% CI:0.4–3.0), 1.4 (95% CI:0.5–4.0), and 1.1 (95% CI:0.4–3.4), respectively.

**Conclusion:**

SM‐HAP/VAP and KP‐HAP/VAP patients in ICU might have a similar prognosis in mortality, the total duration of the artificial airway and ventilator use, the total length of ICU stay, and hospital stay. The risk factors of SM‐HAP/VAP versus KP‐HAP/VAP might be the artificial airway, ventilator use, gastric tube placement, acid suppressant and antibiotics (especially carbapenem).

## Graphical abstract

We compared the prognosis of SM‐HAP/VAP versus KP‐HAP/VAP in the intensive care unit (ICU) and found there was no differences between the 28‐days mortality, the total duration of the artificial airway and ventilator use, the total length of ICU stay, and hospital stay. Besides, we found some risk factors of SM‐HAP/VAP versus KP‐HAP/VAP.

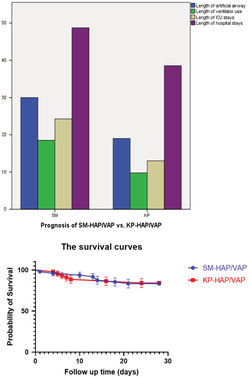

## 1 INTRODUCTION

Hospital‐acquired pneumonia (HAP) is defined as pneumonia that occurs 48 h or more after admission, which was not incubating at the time of admission. Ventilator‐associated pneumonia (VAP) refers to pneumonia that arises more than 48–72 h after endotracheal intubation.^1^, ^2^ HAP is the second most common nosocomial infection in the United States of America^3^ (after urinary tract infections), occurring in five to 10 patients per 1000 hospital admissions.^2^ Up to 6.8% of patients admitted to intensive care units (ICUs) may develop nosocomial pneumonia.^4^ Several pathogens have been reported to cause pneumonia in hospitalized patients, generally involving various bacteria, viruses, and fungi, with an ever‐growing list.^2^, ^5^ VAP occurs in 9–27% of all intubated patients in ICU patients, nearly 90% of episodes of HAP occur during mechanical ventilation.^2^

*Stenotrophomonas maltophilia* (SM) is an environmental bacterium of the *Gammaproteobacteria* class noted in broad‐spectrum life‐threatening infections among vulnerable patients.^6^ SM has been found to cause HAP and is increasingly discovered in the ICU.^7^ A study published by Ibn Saied et al.^8^ found that the independent risk factors for SM‐VAP were ureido/carboxypenicillin or carbapenem exposure the week before VAP, and scores >2 in the respiratory and coagulation components of the Sequential Organ Failure Assessment before VAP. As SM has a natural resistance to many commonly used antibiotics, such as carbapenems and aminoglycosides,^5^ the treatment of SM‐HAP is challenging.

*Klebsiella pneumonia* (KP) is a Gram‐negative pathogen of the *Gammaproteobacteria* class. KP has a large accessory genome of plasmids and chromosomal gene loci.^9^ KP often colonizes the human respiratory, urinary, and intestinal tracts and is an opportunistic pathogen that commonly affects immunosuppressed patients and causes nosocomial infections.^9^ Over the past decade, KP has arisen as a major clinical and public health hazard due to the increasing number of healthcare‐associated infections caused by multidrug‐resistant strains that produce extended‐spectrum β‐lactamases and/or carbapenemases.^10^ Hypervirulent KP can cause serious, rapidly progressing, life‐threatening community‐acquired infection even in young, healthy hosts and has become an important threatening pathogen to human health.^11^

In recent years, many studies have analyzed the risk factors between SM infection and non‐SM infection, but little research compared the prognosis between SM‐HAP/VAP and KP‐HAP/VAP in the ICU. Therefore, the present study aimed to compare the prognosis of SM‐HAP/VAP and KP‐HAP/VAP in the ICU.

## 2 METHODS

### 2.1 Study design and population

This retrospective cohort study included all of the patients who got SM‐HAP/VAP and KP‐HAP/VAP between June 2019 and June 2021 in author's ICU, Shanghai, China, and which was a general comprehensive ICU. The inclusion criteria were (1) ≥18 years of age, (2) patients with SM or KP in their sputum culture during their ICU stay, and (3) patients with HAP/VAP. The exclusion criteria were (1) patients with pneumonia transferred from elderly care homes or other hospitals, (2) incomplete data, or (3) Patients who died of causes other than HAP/VAP within 28 days follow‐up.

This study was approved by the author's hospital. The requirement for informed consent was exempted because it was a retrospective cohort study.

### 2.2 Data collection

Data including age, sex, comorbidities, trauma, tumor, long‐term hormone use, history of immunosuppressive diseases, immunosuppressive drug use, APACHE II score,^12^ Glasgow coma score (GCS),^13^ hemoglobin, bilirubin, creatinine, albumin, oxygenation index,^14^ surgical histories, the duration of gastric tube placement, acid suppressant use, ≥3 antibiotics used, antibiotics duration, carbapenem exposure, the duration of ventilator used, the duration of an artificial airway, and duration of carbapenem before HAP/VAP were collected from the clinical recorders.

### 2.3 Outcomes

The primary outcome was 28‐day mortality. The secondary outcomes were the total duration of an artificial airway, the total duration of ventilator use, the total length of ICU stay, and the total length of hospital stay.

### 2.4 Statistical analysis

All statistical analysis was performed using SPSS 23.0 (IBM, Armonk, NY, USA). Categorical variables were expressed as numbers (percentages) and compared using the chi‐square test or Fisher's exact test. The normality of the distribution of the continuous variables was checked graphically. The continuous variables with a normal distribution were expressed as mean ± standard deviations and tested using the independent samples *t* test. The continuous variables with a skewed distribution were expressed as the median (quartile) (IQR) and analyzed using the Mann–Whitney *U* test. The Cox proportional hazards model was used to estimate hazard ratios (HR) and 95% confidence intervals (CI) for the association between the two species of infection and 28‐day mortality, the total duration of an artificial airway, the total duration of ventilator use, the total length of ICU stays, and the total length of hospital stays. In order to adjust for potential confounders, four multivariable models were used, with progressive degrees of adjustment. The first model was adjusted for age, male, and comorbidities. The second model was further adjusted for creatinine, albumin, and oxygenation index. The third model was further adjusted for the duration of gastric tube placement, duration of acid suppressant use, duration of an artificial airway, and duration of ventilator use. The fourth model was further adjusted for ≥3 antibiotics, carbapenem exposure rate, and duration of antibiotics at baseline. The proportional hazards assumption was checked by plotting the Kaplan–Meier curve and using Schoenfeld residuals. Two‐tailed *P* values <0.05 were considered significant.

## 3 RESULTS

**FIGURE 1:**
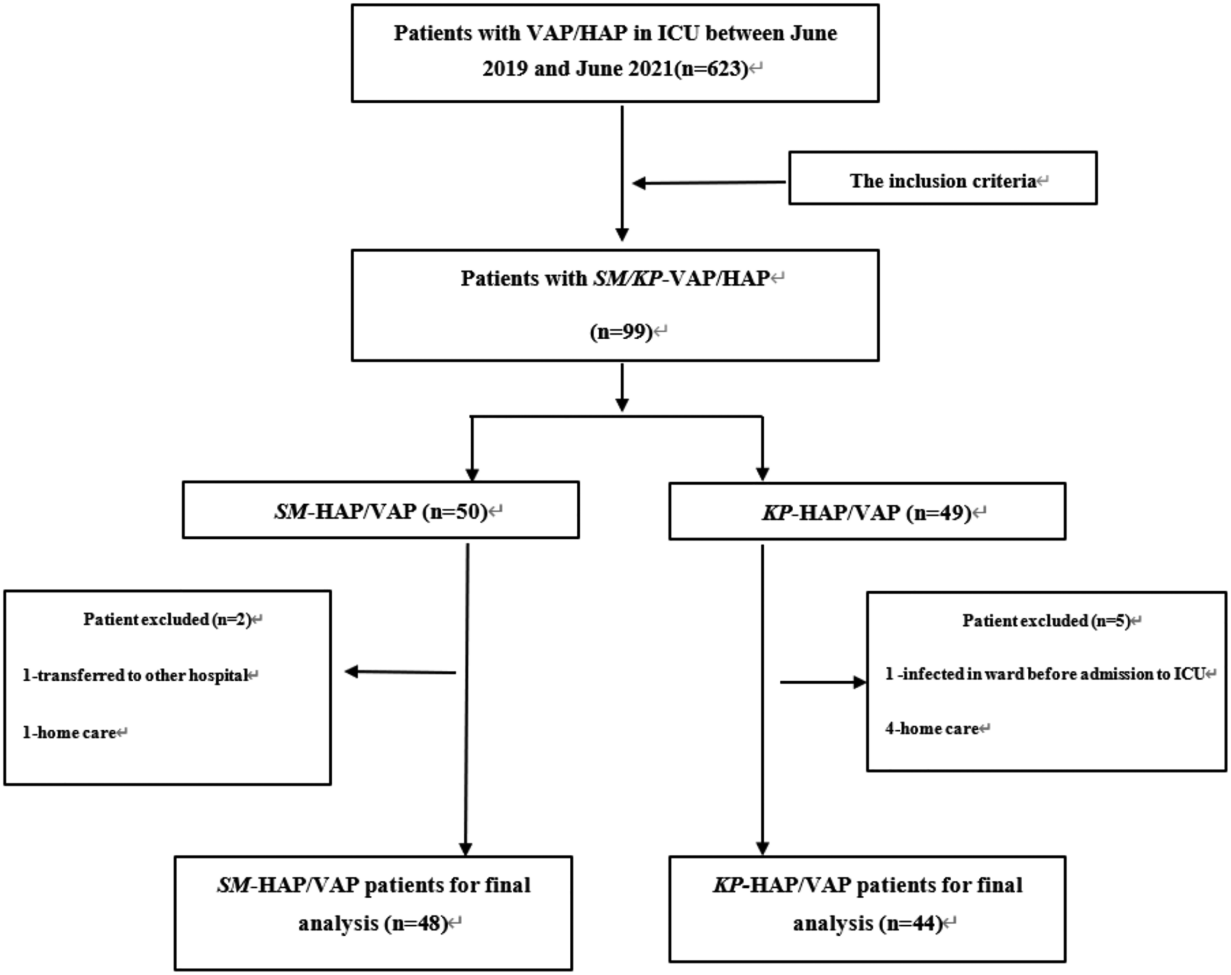
Flow chart of the inclusion of the patients presenting with SM‐HAP/VAP and KP‐HAP/VAP

A total of 92 patients with SM‐HAP/VAP or KP‐HAP/VAP were selected, including 48 with SM‐HAP/VAP and 44 with KP‐HAP/VAP. The patient flowchart is shown in Figure 1. There were no significant differences in the baseline, including age, sex, ≥3 comorbidities, trauma, tumor, hormone use, immunosuppressive diseases/drugs, APACHE II score, Glasgow coma score, hemoglobin, bilirubin, creatinine, albumin, surgery history, oxygenation index, and carbapenem exposure rate between the two groups (all *P* > 0.05). However, the duration of gastric tube placement, duration of acid suppressant use, antibiotics ≥3, artificial airway, ventilator used, and the duration of antibiotics in SM groups were all significantly long than in the KP group before HAP/VAP diagnosis (all *P* < 0.05) (Table 1).

**TABLE 1:** Characteristics of the SM‐HAP/VAP and KP‐HAP/VAP cohorts

Characteristics	SM‐HAP/VAP cohort (*n* = 48)	KP‐HAP/VAP cohort (*n* = 44)	*P*
Baseline characteristics			
Age, median (IQR)	66.0 (53.8, 77.8)	64.5 (57.0, 76.8)	0.907
Male (case, %)	33 (68.8)	33 (75.0)	0.506
≥3 comorbidities (case, %)	12 (25.0)	16 (36.4)	0.237
Trauma [case (%)]	18 (37.5)	15 (34.1)	0.733
Tumors [case (%)]	5 (10.4)	3 (6.8)	0.809
Long‐term hormone use [case (%)]	1 (2.1)	0 (0.0)	>0.999
Immunosuppressive diseases or drug use (case, %)	1 (2.1)	0 (0.0)	>0.999
APACHE II score (mean ± SD)	16.5 ± 6.8	17.8 ± 6.8	0.378
Organ function status			
GCS score, median (IQR)	6.0 (5.0, 10.0)	6.0 (4.0, 13.0)	0.875
Hemoglobin (mean ± SD)	111.1 ± 21.9	108.0 ± 23.3	0.514
Bilirubin, median (IQR)	15.0 (11.5, 21.0)	15.0 (10.0, 21.0)	0.781
Creatinine, median (IQR)	63.5 (49.3, 100.0)	68.5 (49.0, 96.8)	0.690
Albumin, median (IQR)	30.0 (24.3, 33.8)	26.0 (20.0, 33.3)	0.118
Oxygenation index, median (IQR)	256.5 (198.0, 347.9)	278.5 (179.5, 350.8)	0.647
Indicators of therapy before HAP/VAP			
Surgery [case (%)]	33 (68.8)	27 (61.4)	0.457
Duration of gastric tube placement, median (IQR)	11.0 (7.0, 15.8)	2.0 (0.0, 6.0)	<0.001
Duration of acid suppressant used, median (IQR)	5.0 (3.0, 12.0)	2.0 (1.0, 4.0)	<0.001
Duration of artificial airway, median (IQR)	9.5 (5.3, 15.8)	1.5 (0.0, 5.0)	<0.001
Duration of ventilator used, median (IQR)	9.5 (5.3, 15.0)	1.5 (0.0, 5.0)	<0.001
Indicators of antibiotics before HAP/VAP			
Antibiotics ≥3, [case (%)]	22 (45.8)	5 (11.4)	<0.001
Carbapenem exposure rate [case (%)]	14 (29.2)	3 (6.8)	0.006
Duration of antibiotics, median (IQR)	11.5 (6.3, 16.8)	1.0 (0.0, 5.0)	<0.001
Prognostics indicators after HAP/VAP			
Length of artificial airway, median (IQR)	20.5 (5.5, 33.8)	13.00 (3.0, 34.8)	0.38
Length of ventilator use, median (IQR)	7.00 (2.3, 16.0)	7.0 (3.0, 11.0)	0.74
Length of ICU stays, median (IQR)	11.0 (7.0, 21.8)	8.0 (2.3, 16.0)	0.17
Length of hospital stays (mean ± SD)	33.23 ± 21.22	26.50 ± 22.09	0.14

Abbreviations: APACHE II, Acute Physiology and Chronic Health Evaluation II; GCS, Glasgow coma scale; HAP, hospital‐acquired pneumonia; ICU, intensive care unit; IQR, interquartile range; KP, *Klebsiella pneumoniae*; SM, *Stenotrophomonas maltophilia*; VAP, Ventilator‐associated pneumonia.

**FIGURE 2:**
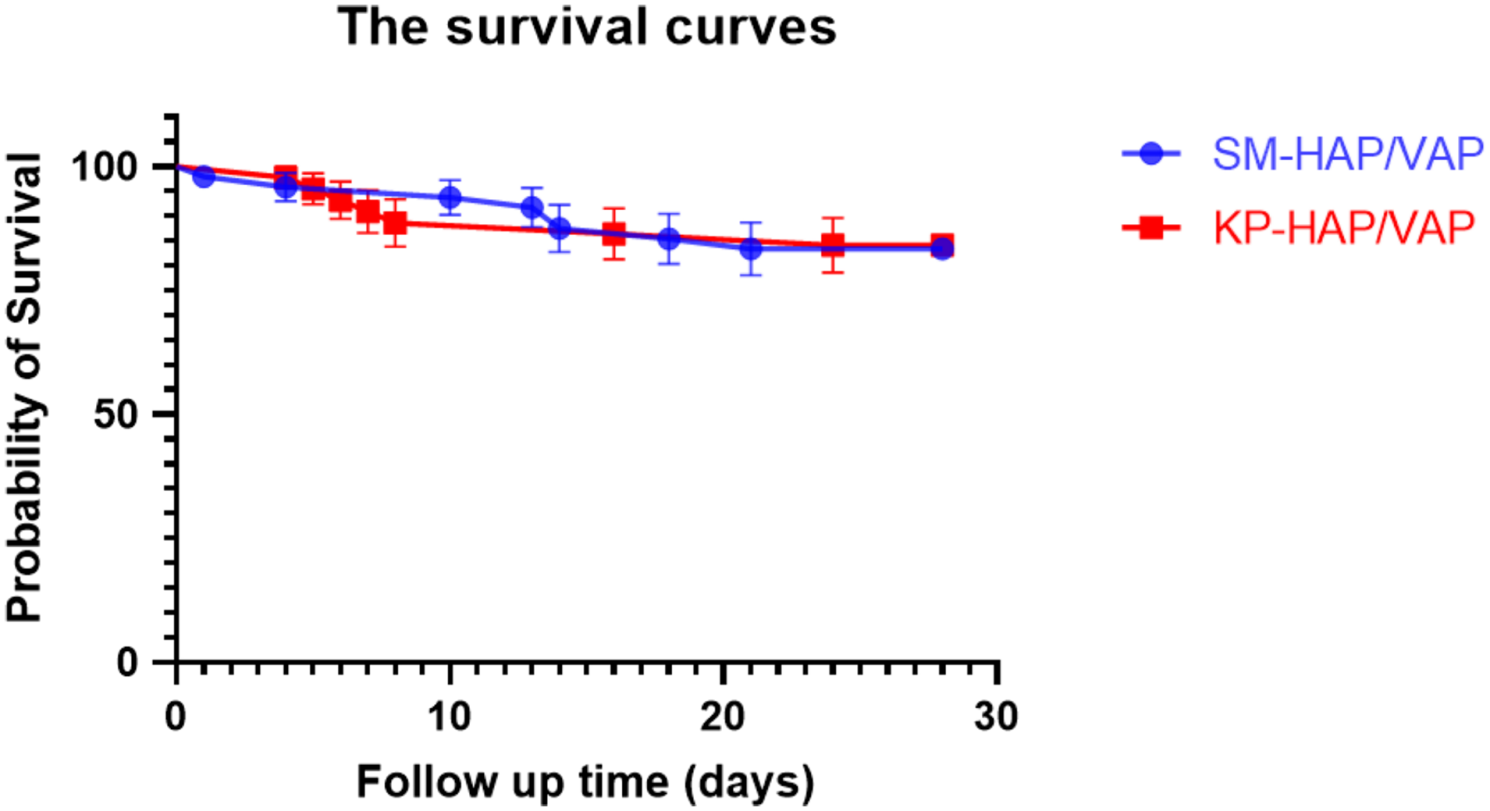
Kaplan–Meier curve of 28‐day mortality by different species of bacterial infection

During the 28‐day follow‐up, 16.7% (8 of 48) of the patients with SM‐HAP/VAP and 15.9% (seven of 44) of the patients with KP‐HAP/VAP died (*P* = 0.922) (Table 2). There was no significant difference in 28‐day mortality between two groups (Figure 2). In a model adjusted for age, male, and comorbidities, the HR for 28‐day mortality comparing SM‐HAP/VAP with KP‐HAP/VAP was 1.3 (95% CI: 0.5–3.7, *P* = 0.602). When further adjusted for creatinine, albumin, and oxygenation index, the HR remained not statistically significant (HR = 1.0, 95% CI: 0.4–3.0, *P* = 0.943); the same was observed after further adjustment for gastric tube placement, duration of acid suppressant use, duration of the artificial airway, duration of ventilator use (HR = 1.4, 95% CI: 0.5–4.0; *P* = 0.535), and after further adjustment for ≥3 antibiotics, carbapenem exposure rate, and duration of antibiotics (HR = 1.1, 95% CI: 0.4–3.4; *P* = 0.847).

**TABLE 2:** Hazard ratio for 28‐day mortality according to SM‐HAP/VAP and KP‐HAP/VAP

	Events/total *n*, %	HR (95% CI)			
		Model 1^a^	Model 2^b^	Model 3^c^	Model 4^d^
28‐day mortality					
SM‐HAP/VAP cohort	8/48 (16.7)	1.3 (0.5–3.7)	1.0 (0.4–3.0)	1.4 (0.5–4.0)	1.1 (0.4–3.4)
KP‐HAP/VAP cohort	7/44 (15.9)	Ref	Ref	Ref	Ref
*P*	0.922	0.602	0.943	0.535	0.847

Abbreviations: CI, confidence interval; HR, hazard ratio.

^a^ Model 1: Adjusted for age, sex, and comorbidities.

^b^ Model 2: Further adjusted for creatinine, albumin, and oxygenation index.

^c^ Model 3: Further adjusted for the duration of gastric tube placement, duration of acid suppressant used, duration of an artificial airway, and duration of ventilator use.

^d^ Model 4: Further adjusted for ≥3 antibiotics, carbapenem exposure rate, and duration of antibiotics.

The total duration of the artificial airway and ventilator use, the total length of ICU stay, and hospital stay in the SM‐HAP/VAP group was similar to these in the KP‐HAP/VAP group (all *P* > 0.05), although they were long in the SM group than in the KP group (Table 1). After we adjusted for four groups of confounders, there was still no statistical difference between them (Table 3).

**TABLE 3:** Hazard ratio for the secondary outcomes according to SM‐HAP/VAP and KP‐HAP/VAP

	Median (IQR)/mean ± SD	HR (95% CI)			
		Model 1^a^	Model 2^b^	Model 3^c^	Model 4^d^
Duration of artificial airway use					
SM‐HAP/VAP cohort	20.5 (5.5, 33.8)	1.1 (0.6–2.1)	1.2 (0.7–2.1)	1.2 (0.7–2.3)	1.5 (0.8–2.9)
KP‐HAP/VAP cohort	13.00 (3.0, 34.8)	Ref	Ref	Ref	Ref
*P*	0.38	0.669	0.552	0.513	0.228
Duration of ventilator use					
SM‐HAP/VAP cohort	7.00 (2.3, 16.0)	1.1 (0.6–2.0)	1.1 (0.6–2.0)	1.1 (0.6–2.1)	1.1 (0.5–2.2)
KP‐HAP/VAP cohort	7.0 (3.0, 11.0)	Ref	Ref	Ref	Ref
*P*	0.74	0.760	0.766	0.700	0.822
Length of ICU stay					
SM‐HAP/VAP cohort	11.0 (7.0, 21.8)	1.4 (0.8–2.6)	1.5 (0.8–2.9)	1.2 (0.6–2.3)	1.1 (0.5–2.1)
KP‐HAP/VAP cohort	8.0 (2.3, 16.0)	Ref	Ref	Ref	Ref
*P*	0.17	0.291	0.179	0.663	0.874
Length of hospital stay					
SM‐HAP/VAP cohort	33.23 ± 21.22	1.1 (0.6–2.0)	1.3 (0.7–2.3)	1.4 (0.8–2.7)	1.8 (0.9–3.5)
KP‐HAP/VAP cohort	26.50 ± 22.09	Ref	Ref	Ref	Ref
*P*	0.14	0.718	0.379	0.260	0.093

Abbreviations: CI, confidence interval; HR, hazard ratio.

^a^ Model 1: Adjusted for age, sex, and comorbidities.

^b^ Model 2: Further adjusted for hemoglobin, bilirubin, creatinine, albumin, and oxygenation index.

^c^ Model 3: Further adjusted for the duration of gastric tube placement, duration of acid suppressant used, duration of an artificial airway, and duration of ventilator use.

^d^ Model 4: Further adjusted for ≥3 antibiotics, carbapenem exposure rate, and duration of antibiotics.

## 4 DISCUSSION

SM‐HAP/VAP and KP‐HAP/VAP patients in ICU might have a similar prognosis in mortality, the total duration of the artificial airway and ventilator use, the total length of ICU stay, and hospital stay.

VAP caused by SM is associated with high morbidity and mortality.^15^, ^16^ However, there was no significant difference in 28‐day mortality between the two groups in this study, and the same conclusion was reached after adjusted for confounding factors. Ibn Saied et al.^8^ found that there was no difference in 30‐day mortality, but 60‐day mortality was higher in patients with SM‐VAP compared to other‐VAP (*P* = 0.056). Mortality could be associated with different therapy strategies. Indeed, Guerci^17^ found that empirical antimicrobial therapy was barely effective while prolonged antimicrobial therapy for more than 7 days and combination antimicrobial therapy had no significant impact on hospital survival in SM‐HAP patients. Adequate treatment, either monotherapy or a combination of antimicrobials, did not modify mortality in SM‐VAP patients versus other‐VAP.^8^

Some data before HAP/VAP onset was collected. After compared them, the present study found some possible risk factors of SM‐HAP/VAP versus KP‐HAP/VAP, and we hope it to be helpful for future research. The present study showed that artificial airway and ventilator use durations before HAP/VAP in the SM group were significantly higher than in the KP group. Patients not walking and suffering from circadian rhythm disorder, sleep deprivation, and absence of family members during ICU stay affect the patients' immune status and increase the risk of SM infection.^18^ Guerci et al.^17^ carried out a retrospective study including all patients admitted to 25 French mixed ICUs between 2012 and 2017 with SM‐HAP during ICU stay and found that SM‐HAP occurred in severe, long‐stay ICU patients who mainly required prolonged invasive ventilation. The longer the ventilator is used, the longer the artificial airway might be, there may be synergies between them. In the present study, the duration of gastric tube placement and duration of acid suppressant were longer in the SM‐HAP/VAP group than in the KP‐HAP/VAP group, as supported by previous studies,^19^, ^20^ but whether they are risk factors of SM‐HAP/VAP versus KP‐HAP/VAP requires more research. Nonetheless, the prophylactic bundle of HAP/VAP is very important in clinical work.^21^, ^22^ In addition, the present study found that the more and the longer antibiotics were used and a higher prior carbapenem exposure were associated with SM‐HAP/VAP, which were confirmed in previous studies.^8^, ^15^ Therefore, we need to control the use of antibiotics as much as possible, especially carbapenems.

In the present study, there were no significant differences in the length of ICU stays and the length of hospital stays between the two groups. There were no significant differences in the length of the artificial airway and the length of ventilator use after HAP/VAP. The respiratory tract is a well‐known source of SM infections, and the clinical response might also be associated with bacterial factors (such as antimicrobial resistance patterns and virulence), patient factors (such as age and comorbidities), and other events that might arise during HAP. SM‐HAP/VAP and KP‐HAP/VAP had a similar outcome, and the following reasons might be responsible for such results. First, once a patient was confirmed to be infected with SM or KP, targeted treatment was conducted according to the drug sensitivity results, and the medication was actively adjusted according to the treatment effect. Second, SM has a low virulence. Third, patients might be infected with highly virulent KP, which could be very difficult to treat, and this study did not identify KP for high virulence. Scholte et al.^23^ found no significant differences in baseline characteristics and duration of mechanical ventilation, length of stay in the ICU and hospital between SM‐HAP/VAP caused by other Gram‐negative bacilli. However, few studies have focused on prognostic indicators other than mortality in SM‐HAP/VAP, so the results need more research to confirm it.

### 4.1 Limitations

There are some limitations. First, this study differs from previous studies regarding patient population, and not enough data are available from the already published studies to compare our outcomes. Second, the study period and the follow‐up were short, and the samples size due to the single participating center might be too small for analysis. Moreover, patients with long‐term home care were excluded. Third, this study corrected for the relevant indicators before HAP/VAP occurrence, but the disease development and treatment effect after infection were not considered. In the future, we will collect relevant therapeutic strategies and other indicators after the diagnosis of HAP/VAP to compare the prognosis of SM‐HAP/VAP and KP‐HAP/VAP. Nevertheless, the literature suggests that a co‐infection of *P* *seudomonas* *aeruginosa* and SM had a synergic impact on the mortality of pneumonia patients.^24^ We did not *record* a co‐infection of SM or other bacteria, so whether they had a synergic impact on mortality is unknown. Hemorrhagic pneumonia is a rare presentation of SM and has 100% mortality within 72 h.^25^ In this study, the final cause of death did not record (such as hemorrhagic pneumonia) either.

## 5 CONCLUSION

In conclusion, regardless of the therapeutic relevance, ICU patients with SM‐HAP/VAP or KP‐HAP/VAP have a similar prognosis, including 28‐day mortality, the total length of ICU stay, hospital stay, the total time of artificial airway, and ventilator use. Further efforts in developing new and active approaches for managing patients with SM or KP are necessary.

## CONFLICT OF INTEREST

The authors declare that they have no conflict of interest.

## ETHICS STATEMENT

This study was approved by the ethics committee of Shanghai Sixth People's Hospital (Approval NO:2021‐KY‐084(K), October 11, 2021). The requirement for informed consent from each patient was waived, because the design of study was retrospective in nature and because of the use of anonymized patient and hospital data.

## AUTHOR CONTRIBUTIONS

Shuping Chen and Dongdong Zou carried out the studies. Dongdong Zou participated in designing and collecting data. Shuping Chen participated in collecting data, performing the statistical analysis, interpreting data, and drafting the manuscript. All authors read and approved the final manuscript.

## Data Availability

The datasets analyzed in this study are not publicly available due to privacy issues.
